# Development of the Double-Blind, Randomized Trials of Effects of Antihypertensive Medicines (DREAM) Database and Characteristics of the Included Trials: Protocol for an Umbrella Review and Meta-Analyses

**DOI:** 10.2196/65205

**Published:** 2025-08-21

**Authors:** Abdul Salam, Rupasvi Dhurjati, Kota Vidyasagar, Prachi Kaistha, Hariprasad Esam, Faraidoon Haghdoost, Rashmi Pant, Amit Kumar, Raju Kanukula, Rakesh Thode, Ravali Baddam, Kanika Chaudhri, Nelson Wang, Soumyadeep Bhaumik, Sonali R Gnanenthiran, Sasi Kumar Tiruttani, Anthony Rodgers

**Affiliations:** 1 The George Institute for Global Health Hyderabad, Telangana India; 2 The George Institute for Global Health University of New South Wales Sydney Australia; 3 Prasanna School of Public Health Manipal Academy of Higher Education Manipal India; 4 School of Medicine Keele University England United Kingdom; 5 The George Institute for Global Health The George Institute for Global Health Sydney Australia; 6 School of Public Health The University of Adelaide Adelaide Australia; 7 Design and Planning School of Architecture The University of Sydney Sydney Australia; 8 Anglia Ruskin University Chelmsford United Kingdom; 9 Cardiovascular Department Royal North Shore Hospital St Leonards Australia; 10 University of Notre Dame Darlinghurst Australia; 11 Sydney Medical School University of Sydney Sydney Australia; 12 School of Population Health University of New South Wales Sydney Australia

**Keywords:** double-blind, randomized controlled trials, blood pressure, anti-hypertensive medicines, systematic review, hypertension, cardiovascular disease, antihypertensive medicines, meta-analyses

## Abstract

**Background:**

A comprehensive evaluation of short-term effects of antihypertensive medicines is important for informing guidelines and clinical practice.

**Objective:**

We aimed to develop the Double-blind Randomized trials of Effects of Antihypertensive Medicines (DREAM) database to facilitate a series of meta-analyses evaluating the short-term effects of antihypertensive medicines.

**Methods:**

We searched the Cochrane Central Register of Controlled Trials, MEDLINE, and Epistemonikos from inception until December 2022 to identify relevant randomized clinical trials (RCTs). We included RCTs in the DREAM database if they were double-blind, enrolled adult participants, evaluated the 5 major classes of antihypertensive medicines over a duration of 2 to 26 weeks, and were published in the English language. Screening of records for inclusion and data collection were both conducted in duplicate. The planned meta-analyses using the DREAM database will follow standard methods as recommended by the Cochrane Handbook for Systematic Reviews. The general methods for these meta-analyses are outlined.

**Results:**

The DREAM database includes 1623 RCTs (4359 comparisons), of which 44% (707/1623) were placebo-controlled, 70% (1141/1623) had parallel-group allocation, and 37% (607/1623) had 3 or more randomized groups. A total of 304,253 participants (mean age 54 years; 509/1623, 46% female) were included, 86% (1391/1623) of RCTs had participants with hypertension, and 11% (175/1623) of RCTs had participants with cardiovascular disease at baseline. RCTs with at least 1 group randomized to combination therapy accounted for 23%(371/1623). The median duration of treatment was 8 weeks. Most (93%, 1509/1623) RCTs reported data on effects on blood pressure.

**Conclusions:**

The first series of meta-analyses using the DREAM database will assess the effects of antihypertensive medicines on blood pressure and safety outcomes, including effects on headache, and cardiovascular events. The findings of these meta-analyses will inform clinical practice guidelines and help identify priorities for future research.

**International Registered Report Identifier (IRRID):**

DERR1-10.2196/65205

## Introduction

High blood pressure (BP) is the leading cause of disease burden globally [1], and antihypertensive medicines are among the most prescribed medicines worldwide to prevent cardiovascular disease events and death. Antihypertensive medicines are also used for the management of other health conditions, including renal disease and migraine. Use of medicines should be guided by comprehensive, up-to-date, and reliable evidence of their safety and efficacy. There is an established collaboration that conducts meta-analyses of individual participant data from randomized controlled trials (RCTs) for long-term effects of antihypertensive medicines [2], and there are many trial-level meta-analyses of the effects of antihypertensive medicines on cardiovascular disease events. However, for short-term efficacy and safety of antihypertensive medicine, the last comprehensive systematic review and meta-analysis was reported 20 years ago [3]. The short-term effects of antihypertensive medicines are important for several reasons. First, guidelines recommend achieving BP control within a few months [4]. Second, maximum BP-lowering effects of antihypertensive drugs accrue within 4 weeks [5] and there is consensus that the extent of BP lowering accounts for most or all cardiovascular event reduction [6,7]. Finally, changes to the antihypertensive medicine regimens are usually done within the first few months [8] of initiation, based on BP levels achieved or adverse events experienced. We, therefore, aimed to develop a database of Double-blind Randomized trials of Effects of Antihypertensive Medicines (DREAM), to facilitate conduct a series of meta-analyses on short-term effects of antihypertensive medicines. The findings of these meta-analyses will inform guidelines, clinical practice, and future research priorities. In this manuscript, we report the methods of development of the DREAM database, present characteristics of RCTs included, and outline the general methods for the planned meta-analyses.

## Methods

### Database Development and Eligibility Criteria for Including RCTs

RCTs were included in the DREAM database if they were randomized, double-blind; enrolled adult participants (age ≥18 years or as defined in individual RCTs); and compared antihypertensive medicines or their combinations with each other or placebo, for a duration of 2 to 26 weeks; reported data to assess the difference in reduction in BP or headache incidence between the groups, given BP lowering efficacy and headache reduction were 2 key areas of interest. Antihypertensive medicines had to be from the 5 major classes of antihypertensive medicines, namely angiotensin-converting enzyme inhibitors, angiotensin II receptor blockers, beta-blockers, calcium channel blockers, and diuretics, and must have been approved by regulatory agencies for hypertension. RCT groups that involved conditional or optional change in therapy (eg, based on BP or adverse effects) such that different participants within a group received different medicines or doses were not included. RCTs among participants with significant health conditions that could alter or interfere with the assessment of the effects of antihypertensive medicines were not included. Also not included were trials published in a non-English language. Detailed inclusion and exclusion criteria, along with the rationale are reported in Table S1 in Multimedia Appendix 1. Additional inclusion and exclusion criteria for the planned individual meta-analyses will be reported in the respective subsequent manuscripts.

### Identification and Selection of RCTs

The following databases were searched using a systematic search strategy (Tables S2, S3, and S4) comprising relevant keywords and MeSH (Medical Subject Headings) terms: Cochrane Central Register of Controlled Trials (until December 2022) for RCTs; and MEDLINE (from 1946 to December 2022), and Epistemonikos (inception to December 2022) for systematic reviews of RCTs. Bibliographies of systematic reviews were reviewed to identify eligible RCTs. In addition, drug approval packages available on the website of the United States Food and Drug Administration were reviewed until December 2022 for unpublished RCTs.

After removing duplicates, 2 reviewers independently assessed in duplicate the title and abstract, and subsequently the full text of each record for assessing eligibility against the eligibility criteria in DistillerSR [9]. Disagreements between the reviewers were resolved by discussion or by adjudication by a third senior reviewer.

### Data Collection

A data collection form was developed to collect characteristics of the RCTs, participants, interventions, and outcomes data, considering the planned analyses. A full list of variables is reported in Table S5 in Multimedia Appendix 1. A data collection guidance document was developed which provided information on where to find the data in the manuscript or report, what data to collect, and if necessary, how to make data conversions. The data collection form and the guidance document were piloted before commencing data collection. For RCTs with more than 1 treatment period, data for all treatment periods were collected. If BP data were reported for more than 1 position in which BP was measured, we collected data for all positions: seated, standing, and supine. Similarly, for the time of BP measurement, data were collected if it was trough or peak. The BP data reported in graphs were extracted in duplicate by 2 reviewers independently using webPlotDigitizer [10] a web-based tool to extract data from images. Reviewers were provided training on data collection. Data from each included RCT were collected in electronic forms using DistillerSR [9]. Conflicts in data collection were resolved by discussion between the reviewers or by adjudication by a third reviewer.

### Planned Meta-Analyses

In the first series of planned meta-analyses, we aim to assess the effects of antihypertensive medicines and their combinations on BP, including evolution or time course of BP-lowering effects, effects on BP variability, safety, effects on headache, cardiovascular events, and death. Where feasible and appropriate, effects will be assessed by medicine class, individual medicines, dose, and participant characteristics at baseline (eg, age, gender, ethnicity, BP, and comorbid conditions). The following are the primary objectives of the first series of planned meta-analyses:

1. To evaluate, among adults, BP-lowering medicines and their combinations compared with placebo for BP-lowering efficacy.
2. To evaluate, among adults, BP-lowering medicines and their combinations compared with placebo for safety.
3. To evaluate, among adults with hypertension (systolic BP [SBP] ≥140 mm Hg, diastolic BP [DBP] ≥90 mm Hg), combinations of BP-lowering medicines as initial therapy compared with placebo, monotherapy, and combinations of other BP-lowering medicines for BP-lowering efficacy and safety.
4. To evaluate, among adults with hypertension (SBP ≥140 mm Hg, DBP ≥90 mm Hg), the time interval at which intensification of BP-lowering medicines is required, if desired BP control is not achieved following initiation or modification of BP-lowering medicines.
5. To evaluate, among adults with hypertension (SBP ≥140 mm Hg, DBP ≥90 mm Hg), BP-lowering medicines and their combinations compared with placebo for effects on cardiovascular events and death.
6. To evaluate, among adults, BP-lowering medicines and their combinations compared with placebo, other BP-lowering medicines, and their combinations for effects on headache.
7. To evaluate, among adults, BP-lowering medicines and their combinations compared with placebo for effects on BP variability.

### Risk of Bias in Included RCTs

For individual meta-analyses, risk of bias in the included RCTs will be assessed using the Cochrane Risk of Bias Tool, or a similar standard tool. Where appropriate, we will use the results of the risk of bias assessment for sensitivity analysis.

### Governance and Data Management Policies

The database is hosted by The George Institute for Global Health. The core team plans to periodically update the database, plan and conduct meta-analyses and disseminate the findings. Other than the planned meta-analyses listed in this manuscript as initial outputs, colleagues, collaborators, and external stakeholder might suggest additional meta-analyses to the DREAM database core team. If appropriate, after approval by the core team, the proposers can work with the core team to develop the protocol, conduct the meta-analyses, and disseminate findings.

### Patient and Public Involvement

This manuscript describes the development of a database for the purpose of a systematic review and outlines secondary analyses, such as evidence generation from published data. Patients were not involved in conceptualizing the DREAM database. For the planned and future meta-analyses, relevant stakeholders will be involved at appropriate stages of the conduct of the meta-analysis to ensure that the results produced are relevant and meaningful to patients affected by the health condition under study, and to those who use evidence to inform guidelines and practice.

### Statistical Analysis

For each planned meta-analysis, a separate protocol will be written, eligible RCTs will be retrieved from the DREAM database, and included in the meta-analysis. Below, we provide an overview of the general methods for the planned meta-analyses.

#### Measures of Treatment Effect

For continuous outcomes, such as change in BP, creatinine, the treatment effect (TE) will be the difference in mean change or difference in standardized mean change (when different scales were used to assess the same outcome values) from baseline to follow-up between the treatment groups. If baseline values are not available, then the difference between mean follow-up values will be used to calculate the TE. When relevant, raw TE will be standardized to the mean values at baseline to adjust for baseline differences between the treatment groups. For dichotomous outcomes (eg, incidence of adverse events), TE will be odds ratios, relative risks, or risk differences between the comparison groups.

#### Unit of Analysis Issues

Treatment groups with same medicine but different doses will be treated as separate groups for analyses of the dose-response effect. Treatment groups with the same medicine, same dose, but different formulations (eg, sustained release) will be combined. When dealing with RCTs involving more than 2 groups and contributing multiple comparisons to the same analysis, the sample size for continuous outcomes and the sample size, as well as the number of patients with the outcome of interest for binary outcomes, in the shared group across contributing comparisons, will be divided by the number of times a group is used to prevent unit of analysis error. Outcome data from multiple follow up time points from the same RCT will not be combined in the same analysis. In a previous large meta-analysis of efficacy of antihypertensive medicines on BP [3], parallel and crossover RCTs yielded similar results and hence will be combined in our analyses, unless they yield significantly different results.

#### Definition of Standard Dose of Antihypertensive Medicines

To facilitate comparisons across medicines, we used an updated standard dose method [3]. The standard dose is defined as the average maintenance dose per day based on the World Health Organization daily defined dose and regulatory approved strengths (Table S6 in Multimedia Appendix 1).

#### Dealing With Missing Data

Data will be assumed to be missing at random. As most RCTs were completed over a decade ago, contacting authors or sponsors to obtain missing data was not feasible. All analyses will be on an intention-to-treat basis, that is, based on the number of participants randomized. If this information is not available, the number of participants who completed the treatment or were included in the analysis will be used. If the sample size for a treatment group is missing, it will be calculated from the overall RCT sample size, splitting it evenly among all groups for parallel RCTs with 1:1 randomization. For cross-over RCTs, the sample size for each group will be the same as that of the overall RCT.

Missing SDs will be calculated using established methods [11]. If it is not possible to calculate, SDs will be imputed as per the following hierarchy based on the available data, for SD of the mean change, SD of the last follow-up mean, SD of the baseline mean, mean SD of the mean change from other RCTs of same medicine or class. For dichotomous outcomes, when 2×2 tables are not reported to derive odds ratio and associated variance, we will impute these based on available data following the method suggested by Elbourne et al [12]. For cross-over RCTs, correlation coefficients are important for both continuous and binary outcomes. If they cannot be calculated from the available data, they will be imputed based on data from other RCTs in the DREAM database [11].

#### Meta-Analysis

Principally, the 3 approaches to analyses will be pairwise meta-analysis, network meta-analysis (NMA), and meta-regression modeling, depending on the research question, objectives and availability of data. A narrative overview of RCTs included in the meta-analysis will provide insights regarding the degree of clinical and methodologic homogeneity among included RCTs, thereby helping to explore the assumptions of homogeneity and similarity for pairwise meta-analysis and NMA.

#### Pairwise Meta-Analysis

Where at least 2 RCTs are available, and if appropriate, we will perform pairwise meta-analysis. Fixed-effect model using inverse variance weighting will be used for meta-analysis of both continuous and binary outcomes. However, where appropriate, a random-effects model may be used. Where relevant, both the models will be used with 1 of the 2 used to assess robustness of results as part of sensitivity analysis.

#### Network Meta-Analysis

The DREAM database has 84 antihypertensive medicines, and 120 unique combinations of from the 5 major classes. In the proposed meta-analyses, where feasible and appropriate, a comparison of 2 or more medicines, classes, or doses will be conducted. NMA is a useful approach for providing a multiway comparison of treatment effects. NMA uses both direct and indirect comparisons to compare multiple (3 or more) treatments simultaneously while maintaining internal randomization in an RCT. It facilitates the ranking of treatments based on parameters of efficacy and safety [13]. NMA will be considered if there is limited direct evidence of comparative effects of pairs of interventions from RCTs, and ranking of interventions for efficacy or safety has important implication for clinical practice and policy. It will be ensured that RCTs contributing to each network are not substantially heterogeneous and there is an absence of important intransitivity, NMA relies heavily on transitivity to draw valid conclusions from indirect comparisons. NMA will be undertaken using either a frequentist or Bayesian framework based on the objectives of the analysis and availability of data. Approaches such as mean rank, median rank, and cumulative ranking probabilities will be used where relative ranking to identify the best of the interventions for a particular outcome is sought. Disagreement between direct and indirect evidence (inconsistencies/incoherence) in a network of interventions will be explored.

#### Meta-Regression

Meta-regression will be conducted, where appropriate, to assess the effects of continuous as well as categorial explanatory variables on the outcome variable. Relatively common outcomes such as BP-lowering will be analyzed with a linear, nonlinear or log-binomial model depending on the type of variable (continuous or categorical). Uncommon events such as rare adverse effects or those occurring over variable periods of time will be studied with a Poisson model. For example, the meta-analysis of time-course of BP-lowering of effect, will be modeled using meta-regression, with medicine classes and monotherapy versus combination therapy as covariates. Bubble plots with 95% confidence bands will be used to visualize the meta-regression plots. Both unadjusted and adjusted analyses will be conducted. Initially, pooled effect estimates with 95% CI will be obtained using unadjusted meta-regression analysis, weighted for RCT sample size. The analysis may be repeated using a multivariate meta-regression model, with adjustment for other covariates including RCT design (parallel or crossover), duration (weeks), baseline mean (mm Hg), and medicine dose.

### Assessment of Statistical Heterogeneity

In evidence synthesis, assessing statistical heterogeneity is essential for ensuring validity, reliability, and interpretability of the generated evidence. The basic way of assessing statistical heterogeneity in results across RCTs included in a meta-analysis would be reviewing the overlap of CIs in a forest plot, with poor overlap suggesting heterogeneity. Heterogeneity, which is due to variability in true effect sizes across trials, will be expressed using 95% predication intervals for the summary effects when there are more than ten RCTs in the meta-analysis. The *I*^2^ statistic, which represents percentage of total heterogeneity across included RCTs that is true heterogeneity and not due to sampling error (chance) will also be assessed. *I*^2^ values will be interpreted as unimportant (0% to 40%), moderate (30% to 60%), substantial (50% to 90%), and considerable (75% to 100%) heterogeneity [14].

### Assessment of Nonreporting (Publication Bias)

When there are at least 10 RCTs included in a meta-analysis, an assessment of nonreporting bias will be performed using funnel plots and statistical tests (eg, the Egger test) [15], and imbalance and asymmetry distance statistics, where appropriate [16]. In network meta-analyses, the presence of small-study effects will be assessed by drawing comparison-adjusted funnel plot that accounts for the fact that different RCTs compare different sets of interventions [18.19]. These assessments will be interpreted in combination with the results of heterogeneity and effect measures being used for the meta-analysis [17,18].

### Subgroup Analysis

Where appropriate and possible, subgroup analysis will be prespecified and performed. Subgroups will be based on important characteristics that modify the effects of antihypertensive medicines, such as class of medicine, treatment duration, baseline BP, and participant characteristics. These subgroups will be used to explore heterogeneity in results across included RCTs. Statistical testing will be used to compare results across subgroups if there are enough RCTs in each subgroup. If appropriate, an *I*^2^ statistic (percentage of variation in effect estimates across subgroups that is due to real subgroup differences) will be computed for subgroup differences.

### Sensitivity Analyses

Where necessary, sensitivity analyses will be performed to assess robustness of results according to assumptions, imputed data, RCTs with a high risk of bias, and different statistical models. The results of sensitivity analyses will be reported in a summary table for easy comparison. As much as possible, sensitivity analyses will be prespecified.

### Assessment of the Certainty of Evidence

The assessment of certainty of evidence will be conducted following GRADE (Grading of Recommendations, Assessment, Development, and Evaluation) approach [19]. When applicable, a summary table will be generated using the GRADEpro GDT software. Since all RCTs included in the DREAM database have randomized double-blind design, initially, the certainty of evidence will be graded as high. However, if there are limitations, the certainty of evidence will be appropriately downgraded based on the risk of bias, consistency across studies, directness of evidence, precision of estimates, and potential publication bias, to moderate, low or very low. The approach suggested by Salanti et al [20] and Adriani Nikolakopoulou et al [21] using the CiNeMA tool will be used to assess confidence in the evidence from network meta-analyses.

### Ethical Considerations

This research involves analysis of secondary data from previously published RCTs; therefore, ethical approval was not required. Individual meta-analyses protocols will be registered on registries such as PROSPERO (International Prospective Register of Systematic Reviews) [22] or INPLASY (International Platform of Registered Systematic Review and Meta-analysis Protocols) [23]. The results of the planned meta-analyses will be published with reference to this prespecified protocol. We will share and present findings of meta-analyses at various scientific meetings and through the collaboration’s networks and memberships across professional societies. For reporting individual meta-analysis results, the PRISMA (Preferred Reporting items for Systematic Reviews and Meta-Analyses) statement’s recommendations will be followed [24].

## Results

**Figure 1:**
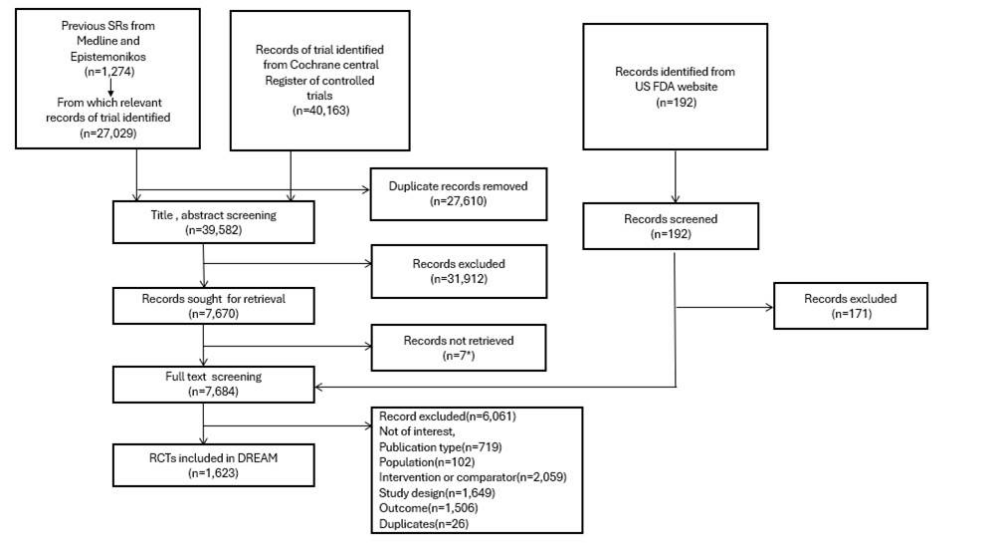
PRISMA (Preferred Reporting Items for Systematic Reviews and Meta-Analyses) flowchart for identification and inclusion of the randomized controlled trials in the Double-blind Randomized trials of Effects of Antihypertensive Medicines database. DREAM: Double-blind Randomized trials of Effects of Antihypertensive Medicines; FDA: Food and Drug Administration; RCT: randomized controlled trial; SR: systematic review.

The PRISMA flow chart (Figure 1) presents the identification and inclusion of RCTs in the DREAM database. A summary of characteristics of included RCT is reported in Table 1. Overall, 1623 RCTs were eligible and included, from which 4359 groups were included in the DREAM database. Most (1096/1623, 68%) RCTs were published before the year 2000. A total of 707 (44%) were placebo-controlled, 482 (30%) were cross-over, and the mean and median duration of intervention were 9 (SD 6) weeks and 8 (IQR 4-12) weeks, respectively.

**Table 1:** Summary of characteristics of randomized controlled trials (N=1623) included in the Double-blind Randomized trials of Effects of Antihypertensive Medicines database.

Characteristics			Randomized controlled trials, n (%)
**Year** **of publication**			
	Before 1990	473 (29)	
	1990 to 1999	623 (38)	
	2000 to 2009	338 (21)	
	After 2009	189 (12)	
**Design**			
	Double-blind	1623 (100)	
	Placebo-controlled	707 (44)	
	Parallel allocation	1141 (70)	
	2 groups	1016 (63)	
	≥3 groups	607 (37)	
**Duration (weeks)**			
	2	63 (4)	
	2.1-4	336 (21)	
	4.1-8	634 (39)	
	>8	590 (36)	
**Funding**			
	Industry	543 (33)	
	Academia, government, nonprofit, or charity	147 (9)	
	Mixed	48 (3)	
	None	4 (0.3)	
	Not reported	881 (54)	
**Sex**			
	Female participants ≥50%^a^	509 (31)	
**Mean age (years)**			
	<50	271 (17)	
	50 to 59	956 (59)	
	60 to 69	185 (11)	
	70 to 79	61 (4)	
	≥80	3 (0.2)	
	Not reported	147 (9)	
**Mean** **systolic blood pressure at baseline (mm Hg)**			
	110-119	12 (0.8)	
	120-129	48 (3)	
	130-139	91 (6)	
	140-149	169 (10)	
	150-159	484 (30)	
	160-169	416 (26)	
	170-179	168 (10)	
	≥180	51 (3)	
	Not reported	184 (11)	
**Condition** **s of interest at baseline**			
	Hypertension^b^	1391 (86)	
	Cardiovascular disease	175 (11)	
	Endocrine conditions	97 (6)	
	Chronic kidney disease	18 (1)	
**Intervention: randomization to at least 1 group**			
	Monotherapy	1545 (95)	
	Angiotensin-converting enzyme inhibitor	432 (27)	
	Angiotensin II receptor blocker	292 (18)	
	Beta-blocker	434 (27)	
	Calcium channel blocker	574 (35)	
	Diuretic	324 (20)	
	Dual combination	354 (22)	
	Triple combination	16 (1)	
	Quadruple combination	1 (0.1)	
**Treatment periods**			
	1	1425 (88)	
	2	150 (9)	
	3	48 (3)	
**Outcomes** **data available**			
	Systolic or diastolic blood pressure	1509 (93)	
	Adverse events data available^c^	809 (50)	

^a^Number of trials in which there were at least 50% female participants.

^b^All participants had hypertension as defined in the individual randomized controlled trials, or trial eligibility clinic systolic BP (SBP) of ≥140 or diastolic BP (DBP) of ≥90 mm Hg or 24-hour ambulatory SBP of ≥130 mm Hg or DBP of ≥80 mm Hg or home SBP of ≥135 mm Hg or DBP of ≥85 mm Hg.

^c^Adverse events data reported: at least 2 groups in a trial reported at least 1 of the following—any adverse events, any serious adverse event, treatment-related adverse event, treatment related serious adverse event, or withdrawals due to adverse event.

Overall, 304,253 participants were included: mean age was 54 years, and in 74% (1205/1623) of RCTs, the participants’ mean age was ≥50 years. Overall, the mean proportion of female participants was 46% (139,182/304,253), and 31% (509/1623) of RCTs had ≥50% female participants. Most RCTs (1391/1623, 86%) had participants with hypertension, and 11% (175/1623) of RCTs had participants with cardiovascular disease. Most common randomization to an antihypertensive medicine class was to calcium channel blockers (at least one group in 574/1623, 35% RCTs randomized to calcium channel blockers), and in 23% (371/1623) of RCTs, at least 1 group was randomized to a combination of 2 or more antihypertensive medicines. Most (1509/1623; 93%) RCTs reported data on effects on systolic or diastolic BP and data for incidence of adverse events were available from 50% (809/1623) of RCTs.

## Discussion

### Principal Findings

The DREAM database is a comprehensive resource of double-blind RCTs, developed to enable a series of meta-analyses on the short-term effects of antihypertensive medicines. These analyses will offer novel insights into the impact of antihypertensive treatments on BP, including its trajectory and variability.

In addition, the database will support evaluations of the safety profiles of these medicines, focusing on their effects on headache and cardiovascular outcomes. Findings will be stratified by drug class, dosage, and key patient characteristics, addressing critical gaps in the current evidence. This comprehensive evidence base will facilitate the development of tools to predict the efficacy of antihypertensive regimens, enabling health care providers to optimize patient care. Ultimately, the findings will contribute to refining clinical practice guidelines, improving treatment strategies, and identifying priorities for future research in the pharmacological management of hypertension and related conditions.

### Comparison With Previous Work

Our work builds on the foundational study by Law et al [3] in 2003, which synthesized 354 randomized double-blind, placebo-controlled trials to evaluate the effects of antihypertensive medicines, including combination therapies. Unlike the analyses by Law et al [3], now over 2 decades old, the DREAM database includes over 1600 double-blind trials published up to 2022, also incorporating active-controlled trials. The work also complements Cochrane Collaboration reviews, which are restricted to specific drug classes. This comprehensive database enables network meta-analyses to assess the relative efficacy and safety of antihypertensive medicines and their combinations, providing updated and robust evidence for clinical decision-making.

This project focuses on short-term (<6 months) effects of these drugs during which most change regimens is usually undertaken, and as such complements meta-analysis of longer-term outcomes. In particular, our work also complements the contributions of the Blood Pressure Lowering Treatment Trialists’ Collaboration [2], which conducts individual patient-level meta-analyses of over 300,000 participants from 100 long-term randomized trials. Blood Pressure Lowering Treatment Trialists’ Collaboration focuses on stratified effects of BP-lowering on major vascular events, all-cause mortality, safety outcomes (eg, renal failure, diabetes, and fractures), and extended effects on conditions such as atrial fibrillation and dementia. Other similar collaborations include the Blood Pressure in Acute Stroke Collaboration and the Beta-Blockers in Heart Failure Collaborative Group, which synthesize evidence to improve cardiovascular outcomes [25]. Together, these efforts strengthen the evidence base for the pharmacological management of hypertension and related conditions, advancing and optimizing cardiovascular care.

### Strengths and Limitations

Our work has several notable strengths. It represents the most comprehensive resource of double-blind randomized trials on antihypertensive medicines to date. The database facilitates stratified meta-analyses by medicine class, dose, and patient characteristics and allows regular updates to enable living systematic reviews providing up-to-date evidence for health care decision-making. However, there are some limitations. Exclusion of trial published in non-English languages and nonavailability of full text of some possibly included RCTs (n=7) despite extensive procurement efforts is a limitation we acknowledge. However, given the small number of these trials, the impact on findings of meta-analyses is likely minimal. The review also includes data abstracted from publication figures, and imputation of data for missing or implausible variability data using standard methods. The lack of individual participant data means we did not have an opportunity to assess in-depth effects across important subgroups of patients based on age, gender, race, and concomitant conditions. Also, given the large number of trials in many countries and a long time frame, safety data were often reported in different ways, hence making overall synthesis more challenging.

## Appendix

Multimedia Appendix 1 Additional material.

## Abbreviations

BP: blood pressure
DBP: diastolic blood pressure
DREAM: Double-blind Randomized trials of Effects of Antihypertensive Medicines
GRADE: Grading of Recommendations, Assessment, Development, and Evaluation
INPLASY: International Platform of Registered Systematic Review and Meta-analysis Protocols
MeSH: Medical Subject Headings
NMA: network meta-analysis
PRISMA: Preferred Reporting Items for Systematic Reviews and Meta-Analyses
PROSPERO: International Prospective Register of Systematic Reviews
RCT: randomized controlled trial
SBP: systolic blood pressure
TE: treatment effect

## Data Availability

The datasets that will be generated during the analysis will be available from the corresponding author on reasonable request.
