# Supplementary material for: Development of the Double-Blind, Randomized Trials of Effects of Antihypertensive Medicines (DREAM) Database and Characteristics of the Included Trials: Protocol for an Umbrella Review and Meta-Analyses
Source: JMIR Res Protoc. 2025 Aug 21;14:e65205. doi: 10.2196/65205 (PMC12411787; doi:10.2196/65205)
Supplement: Multimedia Appendix 1 [file resprot_v14i1e65205_app1.docx]

**Methods of Development Double-blind, Randomised Trials of Effects of Antihypertensive Medicines (DREAM) Database, Characteristics of Included Trials, and Umbrella Protocol for Planned Meta-analyses**

**Supplementary File**

STable 1: Eligibility criteria for the inclusion of RCTs in the DREAM database

| **PICO** | **Inclusion criteria** | **Exclusion criteria** | **Rationale for inclusion/exclusion** |
| --- | --- | --- | --- |
| **Participants** | 1. Adult (age ≥18 years or as defined by the RCT) | 1. Acute/unstable cardiovascular conditions: hypertensive crisis/urgency, acute myocardial infarction (MI), recent MI (within <1 month), acute coronary syndrome, unstable angina, acute stroke, acute heart failure 2. Renal diseases: nephrotic syndrome, dialysis dependent renal failure, IgA-Nephropathy, acute renal failure 3. Severe liver disease 4. Mental illnesses/neurological disorders: acute schizophrenia, acute mania etc., epilepsy, seizers, tremor, and Parkinson’s disease 5. Recent major surgeries/transplants 6. Endocrine disorders (e.g., hyperthyroidism and gigantism) except diabetes 7. Pregnancy, pre-eclampsia 8. Portal hypertension, pulmonary hypertension 9. Benign prostatic hyperplasia 10. Raynaud's disease | 1. Exclusion condition may alter the effects of or influence the assessment of efficacy and/or safety Antihypertensive medicines |
| **Intervention and Comparators** | 1. Antihypertensive medicine(s) from five major classes (ACEIs, ARBs, BBs, CCBs, diuretics) taken orally, compared with placebo or medicine(s) from the same five major classes taken orally 2. Antihypertensive medicines should have WHO’s daily defined dose (DDD) or regulatory (FDA, MHRA or EU country) approved strength. List of included medicines is reported in Supplement S**Table 6**) 3. Treatment period: 2-26 weeks | 1. Concomitant differential treatment between RCT groups with medicines other than those from the five major classes or with non-pharmacological therapy 2. Conditional or optional titration of Antihypertensive medicines such that different participants within a treatment group receive different medicine(s)/dose(s) | 1. The five major classes of Antihypertensive medicines are the mainstay of pharmacological therapy of high BP, they are recommended by hypertension management guidelines as the main classes of medicines, and the effects of these classes on CVD events prevention are established 2. Medicines that do have a WHO DDD or regulatory approved strength are likely to experimental medicines. 3. In case of concomitant interventions/therapies other than the five major classes of Antihypertensive medicines, they should be balanced between the comparison groups such that the effects of five major classes of Antihypertensive medicines can be delineated 4. To allow the evolution of most or full BP lowering effects, a minimum of 2 weeks is necessary. RCTs of long-term treatment period (>26 weeks) usually focus on cardiovascular events and mortality outcomes and are likely to have low adherence to Antihypertensive medicines |
| **Outcomes** | 1. RCTs reporting data for assessing at least one of the following outcomes of difference in,  - change in SBP - change in DBP - BP control - headache incidence | 1. Outcome measurement immediately after exercise, altitude induced and cold induced BP 2. No outcome data for fixed dose treatment periods | 1. Exercise, altitude and cold significantly influences BP |
| **RCT design** | 1. Randomised 2. Double-blind | 1. Cluster/step-wedge RCTs 2. Subgroup analyses, post-hoc analyses, interim reports of RCTs | 1. Double-blind is important for mitigating the risk of bias due to deviations from the intended interventions and the assessments of outcomes 2. Cluster RCTs are usually pragmatic RCTs of strategies rather than efficacy or safety of Antihypertensive medicines |
| **Language** | 1. RCTs reported in English language | 1. RCTs reported in non-English language | 1. There is no evidence suggesting systematic bias from the exclusion of RCTs published in non-English language^2^. Relative proportion of such RCTs would be very small. |

STable 2: Search Strategy for RCTs: EBM Reviews - Cochrane Central Register of Controlled RCTs

| **#** | **Searches** |
| --- | --- |
| 1 | exp Angiotensin-Converting Enzyme Inhibitors/ |
| 2 | (alacepril or altiopril or ancovenin or benazepril or captopril or ceranapril or ceronapril or cilazapril or deacetylalacepril or delapril or derapril or enalapril or epicaptopril or fasidotril or fosinopril or foroxymithine or gemopatrilat or idapril or imidapril or indolapril or libenzapril or lisinopril or moexipril or moveltipril or omapatrilat or pentopril$ or perindopril$ or pivopril or quinapril$ or ramipril$ or rentiapril or saralasin or s nitrosocaptopril or spirapril$ or temocapril$ or teprotide or trandolapril$ or utibapril$ or zabicipril$ or zofenopril$ or Aceon or Accupril or Altace or Capoten or Lotensin or Mavik or Monopril or Prinivil or Univas or Vasotec or Zestril or Enalaprilat).tw. |
| 3 | exp Angiotensin Receptor Antagonists/ |
| 4 | (abitesartan or azilsartan or candesartan or elisartan or embusartan or eprosartan or forasartan or irbesartan or KT3-671 or losartan or milfasartan or olmesartan or saprisartan or tasosartan or telmisartan or valsartan or zolasartan).tw. |
| 5 | exp calcium channel blockers/ |
| 6 | (amlodipine or aranidipine or barnidipine or bencyclane or benidipine or bepridil or cilnidipine or cinnarizine or clentiazem or darodipine or diltiazem or efonidipine or elgodipine or etafenone or fantofarone or felodipine or fendiline or flunarizine or gallopamil or isradipine or lacidipine or lercanidipine or lidoflazine or lomerizine or manidipine or mibefradil or nicardipine or nifedipine or niguldipine or nilvadipine or nimodipine or nisoldipine or nitrendipine or perhexiline or prenylamine or semotiadil or terodiline or tiapamil or verapamil or Cardizem CD or Dilacor XR or Tiazac or Cardizem Calan or Isoptin or Calan SR or Isoptin SR Coer or Covera HS or Verelan PM or azelnidipine or clevidipine).tw. |
| 7 | (aliskiren or ciprokiren or ditekiren or enalkiren or remikiren or rasilez or tekturna or terlakiren or zankiren or ketanserin).tw. |
| 8 | (methyldopa or alphamethyldopa or amodopa or dopamet or dopegyt or dopegit or dopegite or emdopa or hyperpax or hyperpaxa or methylpropionic acid or dopergit or meldopa or methyldopate or medopa or medomet or sembrina or aldomet or aldometil or aldomin or hydopa or methyldihydroxyphenylalanine or methyl dopa or mulfasin or presinol or presolisin or sedometil or sembrina or taquinil or dihydroxyphenylalanine or methylphenylalanine or methylalanine or alpha methyl dopa or guanfacine or guanabenz or guanadrel or guanethidine or debrisoquine or betanidine or guanoxan or guanoclor or guanazodine or guanoxabenz).tw. |
| 9 | (reserpine or serpentina or rauwolfia or serpasil).tw. |
| 10 | (clonidine or adesipress or arkamin or caprysin or catapres$ or catasan or chlofazolin or chlophazolin or clinidine or clofelin$ or clofenil or clomidine or clondine or clonistada or clonnirit or clophelin$ or dichlorophenylaminoimidazoline or dixarit or duraclon or gemiton or haemiton or hemiton or imidazoline or isoglaucon or klofelin or klofenil or m-5041t or normopresan or paracefan or st-155 or st 155 or moxonidine or rilmenidine or rescinnamine or deserpidine or methoserpidine or bietaserpine or azamethonium or mecamylamine).tw. |
| 11 | exp hydralazine/ |
| 12 | (dihydralazine or hydralazin$ or hydrallazin$ or hydralizine or hydrazinophtalazine or hydrazinophthalazine or hydrazinophtalizine or dralzine or hydralacin or hydrolazine or hypophthalin or hypoftalin or hydrazinophthalazine or idralazina or 1-hydrazinophthalazine or apressin or nepresol or apressoline or apresoline or apresolin or alphapress or alazine or idralazina or lopress or plethorit or praeparat hydralazine or diazoxide or minoxidil or nitroprusside sodium or todralazine or tolazoline or endralazine or cadralazine or pinacidil).tw. |
| 13 | exp adrenergic beta-antagonists/ |
| 14 | (acebutolol or adimolol or afurolol or alprenolol or amosulalol or arotinolol or atenolol or befunolol or betaxolol or bevantolol or bisoprolol or bopindolol or bornaprolol or brefonalol or bucindolol or bucumolol or bufetolol or bufuralol or bunitrolol or bunolol or bupranolol or butofilolol or butoxamine or carazolol or carteolol or carvedilol or celiprolol or cetamolol or chlortalidone cloranolol or cyanoiodopindolol or cyanopindolol or deacetylmetipranolol or diacetolol or dihydroalprenolol or dilevalol or epanolol or esmolol or exaprolol or falintolol or flestolol or flusoxolol or hydroxybenzylpinodolol or hydroxycarteolol or hydroxymetoprolol or indenolol or iodocyanopindolol or iodopindolol or iprocrolol or isoxaprolol or labetalol or landiolol or levobunolol or levomoprolol or medroxalol or mepindolol or methylthiopropranolol or metipranolol or metoprolol or moprolol or nadolol or oxprenolol or penbutolol or pindolol or nadolol or nebivolol or nifenalol or nipradilol or oxprenolol or pafenolol or pamatolol or penbutolol or pindolol or practolol or primidolol or prizidilol or procinolol or pronetalol or propranolol or proxodolol or ridazolol or salcardolol or soquinolol or sotalol or spirendolol or talinolol or tertatolol or tienoxolol or tilisolol or timolol or tolamolol or toliprolol or tribendilol or xibenolol or esatenolol).tw. |
| 15 | exp adrenergic alpha antagonists/ |
| 16 | (alfuzosin or bunazosin or doxazosin or metazosin or neldazosin or prazosin or silodosin or tamsulosin or terazosin or tiodazosin or trimazosin or Indoramin or phenoxybenzamine or phentolamine or tolazoline or urapidil).tw. |
| 17 | exp thiazides/ |
| 18 | exp sodium potassium chloride symporter inhibitors/ |
| 19 | (amiloride or benzothiadiazine or bendroflumethiazide or bumetanide or chlorothiazide or cyclopenthiazide or furosemide or hydrochlorothiazide or hydroflumethiazide or methyclothiazide or metolazone or polythiazide or trichlormethiazide or veratide or thiazide?).tw. |
| 20 | (chlorthalidone or chlortalidone or phthalamudine or chlorphthalidolone or oxodoline or thalitone or hygroton or indapamide or metindamideor altizide or bemetizide or benzthiazide or benzylhydrochlorothiazide or butizide or clopamide or epitizide or hydrochlorothiazide or hydroflumethiazide or mefruside or meticrane or metipamide or teclothiazide or tripamide or xipamide or quinethazone).tw. |
| 21 | (azosemide or furosemide or frusemide or fursemide indacrinone or ozolinone or phenoxybenzoic acid or muzolimine or bumetanide or burinex or cicletanine or etozolonie or torsemide or ethacrynic acid or veratide or piretanide or ticrynafen or tienilic acid or tizolemid).tw. |
| 22 | exp Mineralocorticoid Receptor Antagonists/ |
| 23 | (amiloride or triamterene or canrenoate potassium or canrenone$ or spironolactone$ or aldosterone antagonist$ or aldactone$ or practon$ or sc-9420$ or spiractin$ or sc-14266$ or soldactone$ or soludactone$ or aldadiene$ or phanurane$ or sc-9376 or eplerenone$).tw. |
| 24 | exp Antihypertensive Agents |
| 25 | or/1-24 |
| 26 | randomized controlled trial.pt. |
| 27 | random$.tw. |
| 28 | (placebo or ?blind or parallel or cross?over or RCT).tw. |
| 29 | or/26-28 |
| 30 | (abstract or conference or meeting or proceedings or protocol or cluster or letter or comment or editorial or opinion or commentary or process evaluation or non-comparative or single-group or cochrane).ti. |
| 31 | (abstract or conference or letter or comment or editorial or commentary).pt. |
| 32 | (conference or abstract or meeting or scientific).vo. |
| 33 | (mice or rabbit$ or rats or dogs).ti. |
| 34 | or/30-33 |
| 35 | 25 and 29 |
| 36 | 35 not 34 |
| 37 | limit 36 to english language |

STable 3: Search strategy for systematic reviews: Ovid MEDLINE(R)

| **#** | **Searches** |
| --- | --- |
| 1 | exp meta-analysis/ |
| 2 | meta-analy$.tw. |
| 3 | metaanaly$.tw. |
| 4 | exp Meta-Analysis as Topic/ |
| 5 | exp Network Meta-Analysis/ |
| 6 | (systemat$ adj2 review).tw. |
| 7 | pooled analy$.tw. |
| 8 | individual pa$ data.tw. |
| 9 | (individual pa$ data adj3 level).tw. |
| 10 | IPD.tw. |
| 11 | or/1-10 |
| 12 | exp Antihypertensive agents/ |
| 13 | exp Angiotensin-Converting Enzyme Inhibitors/ |
| 14 | ((angiotensin$ or dipeptidyl$ or kininase) adj3 (convert$ or enzyme or inhibit$ or recept$ or block$)).tw. |
| 15 | exp Angiotensin Receptor Antagonists/ |
| 16 | (angiotensin adj3 (receptor antagon$ or receptor block$)).tw. |
| 17 | exp Adrenergic beta-Antagonists/ |
| 18 | (beta adj2 (adrenergic? or antagonist? or block$ or receptor?)).tw. |
| 19 | exp Calcium Channel Blockers/ |
| 20 | (calcium adj2 (antagonist? or block$ or inhibit$)).tw. |
| 21 | exp Thiazides/ |
| 22 | exp Sodium Chloride Symporter Inhibitors/ |
| 23 | (sodium chloride adj (symporter? or cotransporter? or cotransporter?)).tw. |
| 24 | (potassium depleting adj2 diuretic?).tw. |
| 25 | ((loop or ceiling) adj diuretic?).tw. |
| 26 | exp sodium potassium chloride symporter inhibitors/ |
| 27 | (sodium potassium chloride adj2 (cotransporter? or cotransporter? or symporter?)).tw. |
| 28 | exp Mineralocorticoid Receptor Antagonists/ |
| 29 | ((K or potassium) adj sparing adj diuretic$).tw. |
| 30 | exp adrenergic alpha antagonists/ |
| 31 | (adrenergic adj2 (alpha or antagonist?)).tw. |
| 32 | ((adrenergic or alpha or receptor?) adj2 block$).tw. |
| 33 | (renin inhibi$ or renin blocker).tw. |
| 34 | (Centra$ adj2 BP-lowering$).tw. |
| 35 | or/12-34 |
| 36 | 11 and 35 |
| 37 | limit 36 to humans |

STable 4: Search strategy for systematic reviews: Epistemonikos

| title:(antihypertensive*) OR abstract:(antihypertensive*)) OR (title:(hypertens*) OR abstract:(hypertens*)) OR (title:(blood pressure) OR abstract:(blood pressure)) OR (title:(calcium channel) OR abstract:(calcium channel)) OR (title:(beta blocker*) OR abstract:(beta blocker*)) OR (title:(adrenergic) OR abstract:(adrenergic)) OR (title:(Angiotensin) OR abstract:(Angiotensin)) OR (title:(diuretic*) OR abstract:(diuretic*)) OR (title:(thaizide*) OR abstract:(thaizide*)) OR (title:(aldosterone) OR abstract:(aldosterone)) OR (title:(loop*) OR abstract:(loop*)) OR (title:(potassium sparing) OR abstract:(potassium sparing)) OR (title:(renin*) OR abstract:(renin*) |
| --- |

STable 5: Variable for which data was collected from RCTs included in DREAM database

| Trial characteristics |
| --- |
| Trial Name/Acronym |
| NCT/Trial registration number |
| Trial Country/countries |
| Allocation type? |
| Placebo-controlled? |
| Factorial trial? |
| Treatment duration (weeks) |
| Number of randomised treatment groups |
| Participant characteristics and eligibility, background therapy, BP measurement, outcomes |
| Condition at baseline in all participants? |
| All participants were untreated with Antihypertensive medicines for ≥2 weeks immediately before randomisation? |
| All participants were on any standard therapy immediately before randomisation? |
| Details of therapy? |
| Duration (weeks) |
| Source of therapy? |
| Was standard therapy continued during the trial? |
| Eligibility based on BP for randomisation? |
| Reported BP eligibility criteria for randomization? |
| Trial setting? |
| Non-trial drugs or therapies that influence BP were not allowed during the trial? |
| Is BP a primary/main outcome? |
| BP device? |
| Number of BP measurements? |
| Reported BP Outcome Analysis? |
| Disaggregated outcomes by age, sex, race/ethnicity, BMI/weight, diabetes status, CVD status? |
| Number of patients randomised? |
| Age (years) |
| Gender (%) |
| Weight (kgs) |
| Height (cm) |
| BMI (kg/m^2^) |
| Ethnicity (%) |
| Funding body |
| Intervention and baseline BP |
| Intervention name |
| Number of treatment periods? |
| Drug name |
| Treatment duration |
| Types of BP measures? |
| Position of BP measures? |
| Time of BP measures? |
| Measure of variance? |
| Baseline Mean SBP, DBP and its variance (SD, SE or 95% CI) |
| Follow up BP |
| Follow-up week |
| Number analysed |
| Position of BP measures? |
| Time of BP measures? |
| Type of BP data reported? |
| Follow-up mean SBP, DBP and its variance |
| Follow-up Mean change in SBP, DBP and its variance (SD, SE or 95% CI) |
| Difference between groups for SBP, DBP and its variance (SD, SE or 95% CI) |
| BP control percentage and definition of BP control |
| Safety |
| Follow-up week |
| Patients analysed |
| Number of patients with ≥1 any AE |
| Number of patients with ≥1 any SAE |
| Number of patients with ≥1 treatment related AE |
| Number of patients with ≥1 treatment related SAE |
| Number of patients withdrawn/disc. due to any AEs/SAEs |
| Number of patients withdrawn/disc. due to lack of efficacy |
| Number of patients with ≥1 headache event |
| Number of patients with ≥1 CVD event |
| Number of patients with ≥1 dizziness event |
| Number of patients died |
| Number of patients with ≥1 edema event |
| Number of patients with ≥1 cough event |
| Number of patients with ≥1 hypotension event |
| Number of patients with ≥1 hypertension event |
| Biochemical parameters  For the following, data was collected on,   1. Type of data reported.    - 1. Continuous (baseline, mean at the follow-up visit, difference from baseline)      2. categorical (number of people with ≥1 event)      3. Both 2. Units (mmol/L, µmol/L, mEq/L, mg/dL, mg/L, mg/100ml, ml/min, mg%, mL/min/1.73 m^2^) 3. Variance (SD, SE or 95% CI) |
| Sodium |
| Potassium |
| Creatinine |
| Uric acid |
| eGFR |
| Chloride |
| Calcium |

STable 6: Included antihypertensive medicines and their standard dose

| **Number** | **Class and Drug** | **Standard dose** |
| --- | --- | --- |
| *Angiotensin-converting enzyme inhibitors* | | |
| 1 | Benazepril | 5 |
| 2 | Captopril | 50 |
| 3 | Cilazapril | 2.5 |
| 4 | Delapril | 30 |
| 5 | Enalapril | 10 |
| 6 | Fosinopril | 10 |
| 7 | Imidapril | 10 |
| 8 | Lisinopril | 10 |
| 9 | Moexipril | 15 |
| 10 | Perindopril | 5 |
| 11 | Quinapril | 10 |
| 12 | Ramipril | 2.5 |
| 13 | Spirapril | 6 |
| 14 | Temocapril | 10 |
| 15 | Trandolapril | 2 |
| 16 | Zofenopril | 30 |
| *Angiotensin II receptor blockers* | | |
| 17 | Azilsartan | 40 |
| 18 | Candesartan | 8 |
| 19 | Eprosartan | 600 |
| 20 | Fimasartan | 60 |
| 21 | Irbesartan | 150 |
| 22 | Losartan | 50 |
| 23 | Olmesartan | 20 |
| 24 | Telmisartan | 40 |
| 25 | Valsartan | 80 |
| *Beta-blockers* | | |
| 26 | Acebutolol | 400 |
| 27 | Alprenolol | 400 |
| 28 | Atenolol | 50 |
| 29 | Betaxolol | 20 |
| 30 | Bevantolol | 300 |
| 31 | Bisoprolol | 10 |
| 32 | Carteolol | 10 |
| 33 | Carvedilol | 25 |
| 34 | Celiprolol | 200 |
| 35 | Epanolol | 200 |
| 36 | Labetalol | 200 |
| 37 | Metoprolol | 100 |
| 38 | Nadolol | 160 |
| 39 | Nebivolol | 5 |
| 40 | Oxprenolol | 160 |
| 41 | Penbutolol | 40 |
| 42 | Pindolol | 10 |
| 43 | Practolol | 300 |
| 44 | Propranolol | 160 |
| 45 | Talinolol | 100 |
| 46 | Tertatolol | 5 |
| 47 | Timolol | 20 |
| *Calcium Channel Blockers* | | |
| 48 | Amlodipine | 5 |
| 49 | Barnidipine | 10 |
| 50 | Diltiazem | 240 |
| 51 | Efonidipine |  |
| 52 | Felodipine | 5 |
| 53 | Isradipine | 5 |
| 54 | Lacidipine | 4 |
| 55 | Lercanidipine | 10 |
| 56 | Manidipine | 10 |
| 57 | Mibefradil | 75 |
| 58 | Nicardipine | 60 |
| 59 | Nifedipine | 30 |
| 60 | Nilvadipine | 8 |
| 61 | Nimodipine | 300 |
| 62 | Nisoldipine | 20 |
| 63 | Nitrendipine | 20 |
| 64 | Verapamil | 240 |
| *Diuretics* | | |
| 65 | Amiloride | 10 |
| 66 | Bendroflumethiazide | 2.5 |
| 67 | Bumetanide | 1 |
| 68 | Chlorthalidone | 25 |
| 69 | Chlorthiazide | 500 |
| 70 | Clopamide | 10 |
| 71 | Cyclopenthiazide | 0.5 |
| 72 | Eplerenone | 50 |
| 73 | Furosemide | 40 |
| 74 | Hydrochlorothiazide | 25 |
| 75 | Hydroflumethiazide | 50 |
| 76 | Indapamide | 2.5 |
| 77 | Mefruside | 25 |
| 78 | Methyclothiazide | 5 |
| 79 | Metolazone | 5 |
| 80 | Piretanide | 3 |
| 81 | Spironolactone | 50 |
| 82 | Torasemide | 10 |
| 83 | Triamterene | 100 |
| 84 | Xipamide | 20 |

Standard dose: average maintenance dose per day based on WHO daily defined dose and/or regulatory approved strengths.

"Daily defined dose (DDD): The assumed average maintenance dose per day for a drug used for its main indication in adults." “The DDD is sometimes a dose that is rarely or never prescribed because it is an average of two or more commonly used doses.” – WHO

Regulatory approved strengths: strengths ever approved by FDA, MHRA or regulatory agencies of European countries

1. If the DDD was not among the regulatory approved strengths (because DDD is sometimes average of two or more commonly used doses), closest regulatory approved strength that was lower than the DDD was considered as standard dose.
2. For new salts with no DDD, the original salt DDD was considered as standard dose.

**Excluded drugs**

The following 13 drugs were excluded due to the unavailability of WHO Defined Daily Dose and regulatory approved strengths. Altizide, Aranidipine, Azelnidipine, Bemetizide, Canrenone, Dilevalol, Tiapamil, Tripamide, Arotinolol, Benidipine, Bopindolol, Efonidipine, and Gallopamil.
